# Pyrethroids in an AlphaFold2 Model of the Insect Sodium Channel

**DOI:** 10.3390/insects13080745

**Published:** 2022-08-18

**Authors:** Boris S. Zhorov, Ke Dong

**Affiliations:** 1 Department of Biochemistry and Biomedical Sciences, McMaster University, Hamilton, ON L8S 4K1, Canada; 2 Sechenov Institute of Evolutionary Physiology & Biochemistry, Russian Academy of Sciences, Saint Petersburg 194223, Russia; 3 Almazov National Medical Research Centre, Saint Petersburg 197341, Russia; 4 Department of Biology, Duke University, Durham, NC 27708, USA

**Keywords:** pyrethroids, insecticides, sodium channel, channel gating, kdr mutations

## Abstract

**Simple Summary:**

Pyrethroids are a major class of insecticides for controlling arthropod pests and human disease transmitting vectors. Resistance to pyrethroid insecticides presents a serious obstacle to effective pest control. One major mechanism of pyrethroid resistance is caused by mutations in the voltage-gated sodium channel, the target of pyrethroid toxic action. Previous mutational analyses, coupled with homology modeling, predicted two pyrethroid receptor sites on insect sodium channels. Mutations that are located in or close to these receptor sites likely confer resistance by directly reducing pyrethroid binding affinity. However, many mutations appear to be far from the receptor sites, and how these mutations confer pyrethroid resistance remains unknown. In this study, we employed the AlphaFold2 neural network to generate new models of mosquito and cockroach sodium channels. We then used computational methods to dock pyrethroids in the open and closed states of sodium channels to understand the atomic mechanisms of interactions between pyrethroids and sodium channels. Our analysis suggests that mutations residing beyond the receptor sites alter the action of pyrethroids by allosterically affecting the geometry of the receptor sites. The information gained from this study could be valuable for a structure-based design of novel pyrethroids.

**Abstract:**

Pyrethroid insecticides stabilize the open state of insect sodium channels. Previous mutational, electrophysiological, and computational analyses led to the development of homology models predicting two pyrethroid receptor sites, PyR1 and PyR2. Many of the naturally occurring sodium channel mutations, which confer knockdown resistance (kdr) to pyrethroids, are located within or close to these receptor sites, indicating that these mutations impair pyrethroid binding. However, the mechanism of the state-dependent action of pyrethroids and the mechanisms by which kdr mutations beyond the receptor sites confer resistance remain unclear. Recent advances in protein structure prediction using the AlphaFold2 (AF2) neural network allowed us to generate a new model of the mosquito sodium channel AaNav1-1, with the activated voltage-sensing domains (VSMs) and the presumably inactivated pore domain (PM). We further employed Monte Carlo energy minimizations to open PM and deactivate VSM-I and VSM-II to generate additional models. The docking of a Type II pyrethroid deltamethrin in the models predicted its interactions with many known pyrethroid-sensing residues in the PyR1 and PyR2 sites and revealed ligand-channel interactions that stabilized the open PM and activated VSMs. Our study confirms the predicted two pyrethroid receptor sites, explains the state-dependent action of pyrethroids, and proposes the mechanisms of the allosteric effects of various kdr mutations on pyrethroid action. The AF2-based models may assist in the structure-based design of new insecticides.

## 1. Introduction

Pyrethroid insecticides are used extensively to control arthropod pests and human disease-transmitting vectors (WHO, 2007). The toxic effect of pyrethroids is due to their prolonged activation of voltage-gated sodium channels, which are critical for electrical signaling in the nervous system [1,2,3,4,5]. Development of pyrethroid resistance is a major threat to their sustained use in pest and vector control. Knockdown resistance (kdr) to pyrethroids is a well-known mechanism of pyrethroid resistance caused by naturally occurring mutations in sodium channels [5,6,7,8]. In the absence of cryo-EM structures of insect sodium channels within insecticides, the computational docking of pyrethroids aids in the understanding of the atomic mechanisms of their action and resistance.

The pore-forming α-subunit of eukaryotic channels folds from a single polypeptide chain of four homologous domains, I-IV. Each domain contains six transmembrane helical segments (S1–S6). Segments S1 to S4 constitute a voltage-sensing module (VSM). Four VSMs in domains I-IV are connected through linker-helices (S4–S5) to the pore-forming module (PM). The latter contains four outer helices S5, four pore-lining inner helices S6, and four membrane re-entrant P-loops. In the resting channel, the negative membrane potential forces positively charged S4 segments “down” in the cytoplasmic direction. Upon membrane depolarization, the S4 segments move outward which, through linker-helices S4–S5, initiates the opening of the activation gate at the cytoplasmic ends of S6s.

The first model of the pyrethroid-bound sodium channel [9] was elaborated for the house fly open sodium channel, using the crystal structure of a voltage-gated potassium channel Kv1.2 as a template [10]. In this model, pyrethroids bind to the lipid-exposed interface formed by the linker-helix IIS4-S5, the outer helix IIS5, and the inner helix IIIS6. We referred to the first pyrethroid receptor site in the II/III domain interface as PyR1. Later, we elaborated a model of the second pyrethroid receptor site in the I/II domain interface, PyR2, which contains ligand-binding residues in helices IL45, IS5, IS6, and IIS6 [11]. We further proposed that the simultaneous binding of pyrethroids to both PyR1 and PyR2 sites is necessary for the effective prolonging of the sodium channel opening [12].

Molecular modeling was further employed to understand the interactions of pyrethroid with the sodium channels in different insect species. For example, a hypothesis was proposed to explain the preferential binding of acaricidal pyrethroids to the sodium channels of ticks/mites [13]. The docking of permethrin and fenvalerate to the honeybee sodium channel revealed that the PyR1 site is similar to that in other insects [14]. Selective resistance of the bumble bee sodium channel to tau-fluvalinate was explained [15].

Identification of residues that are critical for the binding and action of pyrethroids is valuable not only for understanding the interactions of pyrethroids with sodium channels, but also for resistance monitoring. For example, systematic mutational analysis, coupled with computational modeling, uncovered a V253A mutation in the S4–S5 linker in domain I that reduced mosquito sodium channel sensitivity to pyrethroids [11]. Interestingly, a different substitution, V253F, at the same position was recently detected and concluded to be associated with pyrethroid resistance in an *Ae. aegypti* mosquito population in Brazil [16]. The role of this mutation has recently been functionally confirmed in the Xenopus oocyte system [17].

Due to a limited precision of previous homology models, which are based on the crystal and cryo-EM structures of potassium channels and prokaryotic sodium channels, the mechanisms of the state and dependent action of pyrethroids within and beyond the PyR1 and PyR2 sites are not well understood. Here, we employed the AlphaFold2 (AF2) neural network software to model the mosquito sodium channel AaNav1-1. The AF2 neural network is trained on crystal and cryo-EM structures of transmembrane proteins, including ion channels, in the absence of membrane potential. In the experimental structures of ion channels, VSMs are activated (S4 segments are in the “up” position), and PM is presumably inactivated. As a consequence, the AF2 model of AaNav1-1 predicted the channel with activated VSMs and inactivated PM.

We employed Monte Carlo energy minimizations (MCM) to impose the open PM conformation of the AaNav1-1 channel, as seen in the cryo-EM structure of the open rat Nav1.5 channel [18], docked a Type I pyrethroid permethrin (PMT) and a Type II pyrethroid deltamethrin (DMT) to this model, and found that the ligands are in direct contact with all residues whose kdr mutations within the PyR1 and PyR2 sites are reported. We further inactivated PM and deactivated VSMs in silico with bound DMT and monitored the DMT-bound contacts to understand why pyrethroids stabilize the activated channels. We further analyzed state-dependent contacts of those residues beyond the PyR1 and PyR2 sites, which are subject to kdr mutations, and found that these contacts stabilize the mutual disposition of the channel segments that contribute to the PyR1 and PyR2 sites. We propose that respective kdr mutations would allosterically affect the geometry of the PyR1 and/or PyR2 sites and hence, the affinity of the ligands. Our study integrates available experimental data and explains the state-dependent action of pyrethroids that bind within or beyond the PyR1 and PyR2 sites. The proposed structural models suggest mechanisms by which kdr mutations affect the action of pyrethroids and by which they may assist in the structure-based design of new pyrethroid insecticides.

## 2. Materials and Methods

### 2.1. AlphaFold2 Models

The AlphaFold2 (AF2) neural network software [19] is a cutting-edge computational technology, which in the latest CASP 2020 competition (Critical Assessment of Techniques for Protein Structure Prediction) greatly outperformed other methods in terms of the number of correct predictions and their accuracy [20]. We employed the AF2 software installed at the Digital Research Alliance of Canada (alliancecan.ca) to build full-fledged models of the mosquito sodium channel AaNav1-1 (*Aedes aegypti,* GenBank: AGG53064.1) and the cockroach sodium channel BgNav1-1a (*Blattella germanica*, GenBank: U73583.1). Cytoplasmic N- and C-ends and inter-domain linkers I/II and II/III of sodium channels are known to interact with cytoplasmic proteins, but details of these interactions are unknown or poorly understood. Since the AF2 models ignore the impact of cytoplasmic proteins, the respective cytoplasmic segments are disordered. We removed these segments from the models.

### 2.2. Modeling Channel States with the Open PM and Resting VSMs

We used the cryo-EM structure of rat Nav1.5 channel in the open state [18] to transform the PM of the insect channels in the open state using the methodology of large-scale in silico transformations of PM in full-fledged channel models, as described by the authors of [21]. Voltage-sensing domains were transformed from the activated to resting states, as described in the study in [22]. The designation of different models of the channels is given in Supplementary Figure S1B.

### 2.3. Residue Designations and 3D Alignment of Channel Structures

**Figure 1:**
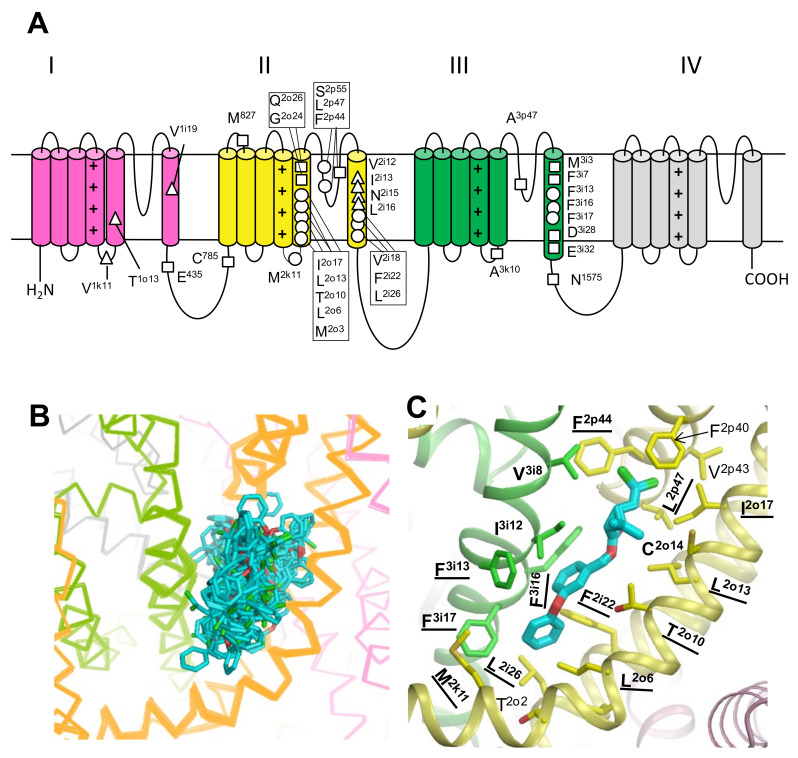
(**A**) Transmembrane topology of voltage-gated insect sodium channels indicating residues within PyR1 (circles) or PyR2 (triangles), or beyond PyR1 and PyR2 (squares), for which kdr mutations have been functionally confirmed in *Xenopus* oocytes. Residues are labeled according to the scheme, which is universal for P-loop channels [11,23]. A residue label includes the amino acid one-letter code, the domain number (I–IV), segment type (*k*, the linker-helix S4–S5; *i*, the inner helix S6; *p*, the P-loop; and *o*, the outer helix S5), and relative number of the residue in the segment. (**B**) Ensemble of PMT starting poses in the PyR1 site of the mosquito sodium channel model with inactivated pore domain, ^i^AaNav1-1. (**C**) A low-energy binding pose of PMT in PyR1. Side chains of residues within 4 Å from PMT are shown as sticks. Labels of residues for which kdr mutations are reported (Table 1) are bold-underlined. Labels of residues for which kdr mutations are unknown, but engineered mutations are shown to affect the action of pyrethroids (Table 2), are bold-typed.

We used a residue-labeling scheme, which is universal for P-loop channels [23], as shown in Figure 1 and Supplemental Figure S1. This labeling scheme highlights symmetric locations of residues in different channel domains. We also used residue numbers in the housefly voltage-sensitive sodium channel (*Musca domestica*, GenBank: AAB47604).

Different models and experimental structures were 3D-aligned by minimizing the root mean square deviations of C^α^ atoms in P1 helices, the most 3D-consereved segments of P-loop channels, by matching atoms in the Kv1.2-Kv2.1 channel (PDB code: 2R9R).

### 2.4. Energy Optimizations, Ligand Docking, and Visualization

Calculations were performed with the ZMM program (http://zmmsoft.ca) that optimizes molecular structures in the space of generalized (internal) coordinates [24,25] using Monte Carlo energy minimizations [26]. Docking of pyrethroids from multiple starting poses into the PyR1 and PyR2 sites was performed as described elsewhere [12,27]. Molecular images were created using the PyMol Molecular Graphics System, Version 0.99rc6 (Schrödinger, LLC, New York, NY, USA).

## 3. Results and Discussions

### 3.1. The AF2 Model of AaNav1-1

Supplemental Figure S2 shows the best-ranking full-fledged AlphaFold2 model of the mosquito sodium channel AaNav1-1 superimposed with the cryo-EM structure of the non-functional cockroach sodium channel NavPaS [28]. The folding of the AF2 model in the transmembrane and extracellular regions is similar to that seen in the cryo-EM structures of sodium channels, which contain the activated voltage-sensing modules (VSMs) and the pore module (PM) in presumably inactivated state [29,30,31]. In addition, the AF2 model predicted several structured segments in cytoplasmic parts of the channel, which are not resolved in the cryo-EM structures of the sodium channels. We further refer to this model ^i^AaNav1-1, where the superscripted prefix ‘i’ indicates the inactivated PM.

We used the ZMM program to calculate the energy of the AF2 model. Since both the AF2 software and the ZMM program employ the AMBER force field (although different versions), the ZMM energy of the ^i^AaNav1-1 model had favorable energy and lacked any clashes. MC-minimization of the ^i^AaNav1-1 model with fixed backbones and elastic bond angles in proline residues further decreased the energy.

We also generated AF2 models of the cockroach sodium channel ^i^BgNav1-1a. The best-ranking AF2 models of the mosquito and cockroach sodium channels have very similar transmembrane parts, but some differences in the C-terminal domain and within a large extracellular loop (Figure S3A,B). These segments are far from the PyR1 and PyR2 sites. Importantly, the PyR1 and PyR2 sites are practically identical in the AF2 models of both channels (Figure S3C,D). Therefore, we further used only the mosquito sodium channel models. The 3D structures of the sodium channels of other insect organisms may deviate from that of AaNav1-1, especially in the extracellular and cytoplasmic loops. However, the fact that many different insect organisms are sensitive to the representative Type I and Type II pyrethroids DMT and PMT suggests that the geometry of the PyR1 and PyR2 sites in these organisms is generally similar. Peculiarities of the PyR1 and PyR2 sites in different organisms may underlie the species-selective action of some pyrethroids [15]. Analysis of these peculiarities would require the AF2 modeling of respective channels and the docking of species-specific pyrethroids. Such analysis is beyond the goals of this study.

Table 1 shows kdr mutations for which respective wild-type residues are within 4 Å from the pyrethroids that we docked into the PyR1 and PyR2 sites, as described below (Section 3.2 and Section 3.3). Table 2 shows engineered mutations within the PyR1/PyR2 sites that were generated and confirmed earlier in functional studies.

### 3.2. Docking of PMT in the PyR1 Site in Model ^i^AaNav1-1

We generated 2000 random starting poses of PMT at the PyR1 site of ^i^AaNav1-1 (Figure 1b), MC-minimized the energy for each starting pose, and collected the ligand-binding poses for which the energy of ligand–channel interactions was within 5 kcal/mol above the apparent global energy minimum. In the collected ensemble, we have chosen the PMT binding pose in which the ligand interacts with the maximal number of experimentally known pyrethroid-sending residues (Figure 1c). These include 12 residues whose kdr mutations are listed in Table 1 and two residues whose engineered substitutions affect the action of pyrethroids in functional studies (Table 2). This binding pose resembles the orientation of bifenthrin and PMT in the Kv1.2-based model of the housefly sodium channel [9,68] and the complex of τ-fluvalinate with the NavMs-based homology model of the open bumble bee sodium channel [15].

### 3.3. Docking of DMT in the PyR1 and PyR2 Sites in Model ^o^AaNav1-1

The sampling strategy illustrated in Figure 1 was used to dock DMT in the PyR1 and PyR2 sites. The ^i^AaNav1-1 model generated above using the AF2 neural network predicts the AaNav1-1 channel structure in the energetically most preferable state, with activated VSMs and presumably inactivated PM. However, pyrethroids are known to stabilize sodium channels in the activated state [5]. Therefore, we transformed ^i^AaNav1-1 into the state with the open PM by stepwise moving C^α^ atoms of transmembrane helices in PM towards positions in the cryo-EM structure of the rat Nav1.5 channel, whose PM is captured in the open state [18]. Superposition of the obtained open-PM model ^o^AaNav1-1 with the starting model ^i^AaNav1-1 is shown in Supplemental Figure S4a,b. The in silico opening of PM in the ^i^AaNav1-1 model caused rather small changes at the PyR1 site (Figure S4c). Therefore, the binding pose of PMT in the PyR1 site of the ^o^AaNav1-1 model is likely similar to that in the ^i^AaNav1-1 model (Figure 1c). However, the PM activation caused significant changes in the PyR2 site, especially in the IIS6 helix with pyrethroid sensing residues, including L^2i16^ that moved closer to the linker-helix IS4-S5 (Figure S4d).

**Figure 2:**
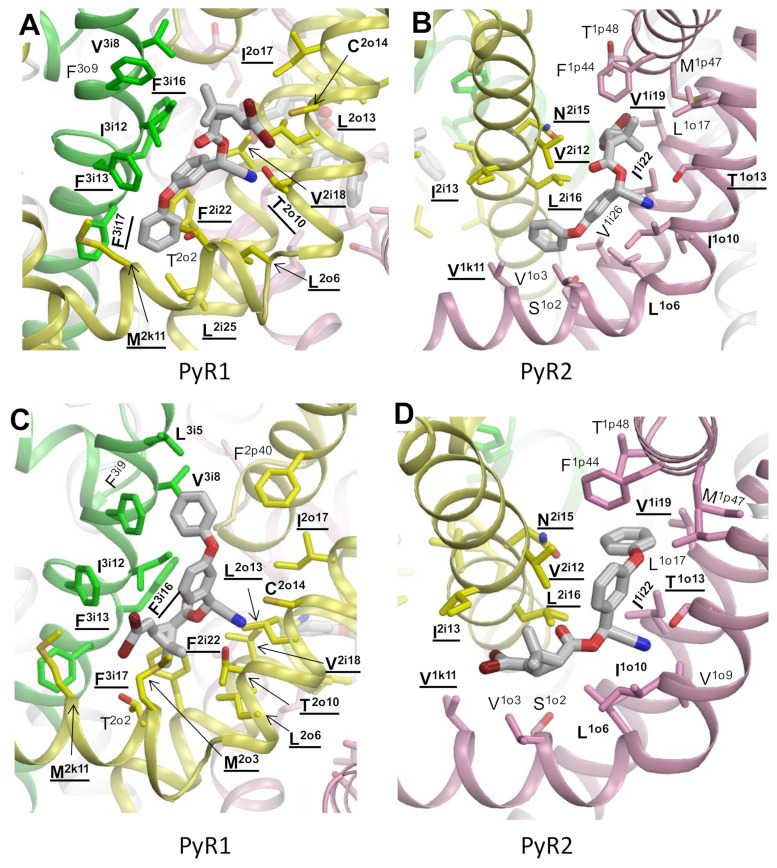
Alternative binding modes of deltamethrin in the PyR1 (**A**,**B**) and PyR2 (**C**,**D**) sites of the mosquito sodium channel with an in silico opened pore domain, ^o^AaNav1-1. Side chains of residues within 4 Å from DMT are shown. Labels of residues for which kdr mutations are reported (Table 1) are bold-underlined. Labels of residues for which kdr mutations are unknown, but the engineered mutations that are shown to affect the action of pyrethroids (Table 2) are bold-typed.

Pyrethroids, particularly Type II pyrethroids, such as DMT, preferably bind to the activated state of insect sodium channels and exhibit strong state-dependent action [70,71]. Therefore, model ^o^AaNav1-1 with the activated VSMs and in silico opened PM was then used to dock DMT in sites PyR1 and PyR2. We performed unbiased docking of DMT in the PyR1 and PyR2 sites from multiple starting poses as described for PMT in the ^i^AaNav1-1a model (Figure 1b). Representative low-energy structures from the collected ensembles of binding poses are shown in Figure 2. In both the PyR1 and PyR2 sites, we found DMT binding poses in two opposite orientations. In one orientation, the dimethylcyclopropane ring fit the corner between the S4–S5 and S5 helices, whereas the terminal aromatic ring deeply penetrated into the fenestrations, as previously predicted [11,12]. In another ensemble, the tricyclic ring penetrated into the fenestrations and the terminal aromatic ring bound between helices S4–S5 and S6, as in models of bifenthrin and PMT in the Kv1.2-based models of the housefly sodium channel [9,68]. The latter orientation is also seen in our model of τ-fluvalinate in the bumble bee channel [15] and in the current AF2-based model of ^i^AaNav1-1 with PMT in the PyR1 (Figure 1c).

In both ensembles, the nitrile group of DMT formed favorable contacts with polar residues in the S5 helices. In the PyR1 site, the bromine atoms approached the sulfur atom either in C^2i14^ (Figure 2a) or in M^2k11^ (Figure 2b), forming favorable bromine–sulfur halogen bonds [72]. In the binding pose shown in Figure 2c, DMT approached L^3i5^. Mutation F^3i5^L substantially decreased binding of several pyrethroids in the bumble bee sodium channel [15]. In our model ^o^AaNav1-1, replacement L^3i15^F would enhance the binding of DMT due to π-stacking with the terminal aromatic ring of the ligand. The major difference between models of PMT in ^i^AaNav1.1 (Figure 1c) and DMT in ^o^AaNav1-1 (Figure 2a) is a favorable interaction of the DMT nitrile group with T^2o10^.

In both orientations of DMT in the PyR2 site, the ligand was with 4 Å from seven residues whose kdr mutations are reported (Table 1). These include two residues, V^1k11^ and T^1o13^, whose kdr mutations were recently functionally confirmed [17]. The energy of ligand–channel interactions in the opposed DMT-binding poses was comparable. We are not aware of experimental data that would enable us to discriminate between these binding poses of DMT and favor one of them.

As compared with the model of ^i^AaNav1-1 with PMT in PyR1 (Figure 1c), the model of ^o^AaNav1.1 with DMT in PyR1 (Figure 2a) has two peculiarities. Firstly, the DMT nitrile group H-bonded to T^2o10^. Secondly, the 2,2-dibromoetheny group in DMT, which is larger and more polarized than the 2,2-dichloroethenyl group in PMT [30], would establish stronger contacts with the channel residues.

### 3.4. In Silico Inactivating ^o^AaNav1-1a with Two DMT Molecules in the PyR1 and PyR2 Sites

To understand how pyrethroids inhibit the channel transition from the open to the inactivated state, we in silico transformed model ^o^AaNav1-1, with two bound DMT molecules (Figure 2a,b), back to the inactivated state by stepwise moving C^α^ atoms in helices S4–S5, S5, and S6 to their positions in the original AF2-based model ^i^AaNav1-1, which we name ^o-i^AaNav1-1.

**Figure 3:**
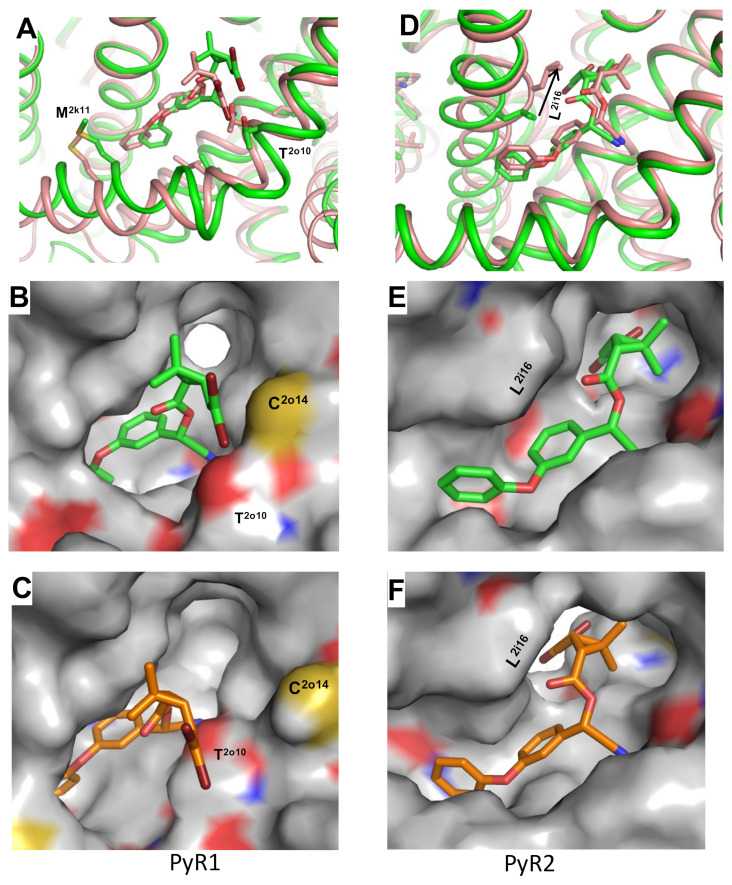
In silico inactivating DMT-bound model ^o^AaNav1-1. (**A**) Superposition of models ^o^AaNav1-1 (green) and ^o-i^AaNav1-1 (pink) with DMT in PyR1. (**B**,**C**) Surface representation of models ^o^AaNav1-1 (**B**) and ^o-i^AaNav1-1 (**C**) with DMT in site PyR1. Note a significant shift of the CBr_2_ group that lost halogen bonds with T^2o10^ and C^2o14^. (**D**) Superposition of models ^o^AaNav1-1 and ^o-i^AaNav1-1 with DMT in site PyR2. (**E**,**F**) Surface representation of models ^o^AaNav1-1. (**E**) and ^o-i^AaNav1-1 (**F**) with DMT in PyR2. Note a significant shift of L^2i16^ that lost favorable contacts with the DMT aromatic ring.

The folding of the in silico inactivated ^o-i^AaNav1-1 model with DMT molecules in the PyR1 and PyR2 sites is very similar to that of the original model ^i^AaNav1-1, with inactivated PM. The linker helix IIS4-S5, which contains M^2k11^, shifted in the cytoplasmic direction and closer to VSM-II (Figure 3a). DMT in the PyR1 site shifted to preserve a contact between its terminal aromatic ring and the linker-helix. The 2,2-dibromoetheny moiety moved farther from the fenestration, which apparently shrunk, and lost its favorable halogen bonds with the sulfur atom of C^2o14^ and the oxygen atom of T^2o10^ (cf. Figure 3b,c). Overall, the favorable DMT contacts with the PyR1 site in ^o^AaNav1-1 would resist the channel transition into the inactivated state, where DMT contacts with ^o-i^AaNav1-1 are weaker. This could be one cause of the preferable binding of pyrethroids to the activated channels.

The major change in the PyR2 site upon the channel inactivation from the open state involves a large shift of L^2i16^ (Figure 3d), which lost its favorable contacts with the aromatic ring of DMT (cf. Figure 3e,f). These transformations may be considered as another cause of preferable binding of pyrethroids to the activated channels. Thus, favorable DMT interactions with the PyR1 and PyR2 sites in the open, but not inactivated, PM contribute to the state-dependent action of pyrethroids.

### 3.5. In Silico Deactivating VSM-I and VSM-II in ^o^AaNav1-1 with DMT in the PyR1 and PyR2 Sites

**Figure 4:**
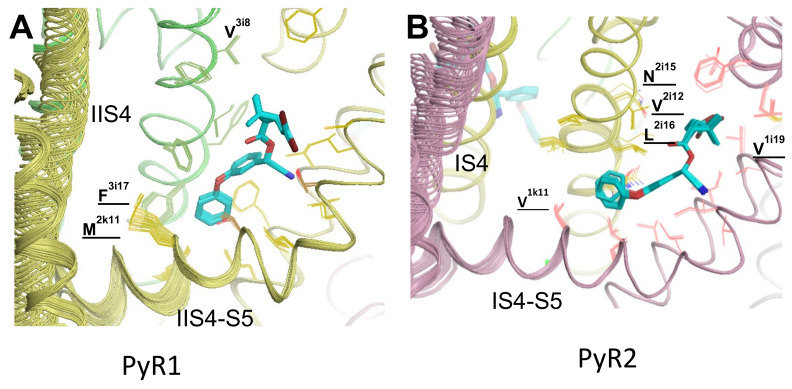
In-silico deactivating VSMs in DMT-bound model ^o^AaNav1-1. (**A**) In site PyR1, the side chain of M^2k11^ moves significantly from DMT. (**B**) In site PyR2, the side chains of I^2i13^ and L^2i16^ move from DMT.

Linker helices IS4-S5 and IIS4-S5, which contain pyrethroid-sensing residues, are immediately connected to the voltage-sensing helices in VSM-I and VSM-II, respectively. To explore the impact of deactivation of VSM-I and VSM-II on the binding of pyrethroids in PM, we in silico moved helices IS4 and IIS4 from the activated “up” positions to the resting “down” positions. The models with the deactivated VSMs are designated ^o-d^AaNav1-1. The computations were performed with two DMT ligands bound to the PyR1 and PyR2 sites, with the aromatic rings in the interfaces between helices S4–S5 and S6, and the dimethylcyclopropane rings in the fenestrations (Figure 4a,c). The voltage-sensing arginines were step-wise shifted in the cytoplasmic direction, and the structure was MC-minimized at each step, as previously described [22]. As expected, the N-ends of the linker helices IS4-S5 and IIS4-S5 shifted down with the S4 helices.

DMT-sensing valine V^1k11^ in the linker helix IS4-S5 of PyR2 [12] and DMT-sensing methionine M^2k11^ [73] in the linker helix IIS4-S5 receded from the DMT molecules. DMT interactions with F^3i17^ in PyR1 and L^2i16^ in PyR2 weakened, while most of other ligand–channel contacts changed insignificantly. Interaction of DMT and PMT with V^1k11^ was predicted by molecular modeling and confirmed in functional studies [12]. Mutation V^267/1k11^F was recently found in pyrethroid-resistant *Ae. aegypti* mosquitoes [16], and the role of this mutation, V^267/1k11^F, in pyrethroid resistance has been functionally confirmed in *Xenopus* oocytes [17]. The weaker contacts of DMT with pyrethroid-sensing residues in the linker helices IS4-S5 and IIS4-S5 could be the third cause of the preferable binding of pyrethroids to the activated channels.

Some kdr mutations within PyR1/PyR2 sites may have dual effect on the action of pyrethroids. The mutations may affect the channel gating and decrease the open-state probability of the channel, as previously reported [70,74,75]. This would directly or indirectly enhance the action of kdr mutations on the ligand binding. For example, several kdr mutations involving V^410/1i19^ are reported: V^410/1i19^M/A/G in *Helicoverpa zea* [32], V^410/1i19^M in *Heliothis virescens* [74], and V^410/1i19^L in *Cimex luctularis* [33]. In our Kv1.2-based model, V^410/1i19^ was rather far from the PyR2 site [12]. In the AF2-based open-state model ^o^AaNav1-1, V^410/^^1i19^ directly interacts with the PyR2-bound DMT in both binding poses (Figure 2b,d). Smaller substitutions V^410/1i19^A/G would decrease these interactions, while larger substitutions V^410/1i19^M/L would repel DMT from its binding site, supporting direct interactions of DMT with V^4101i19^. On the other hand, mutations V^1i19^M/L are known to affect the channel gating [5], likely due to repulsion of the large substitutions from the nearby M^1p47^ (Figure S5c). The repulsion would affect the position of the cytoplasmic half of IS6, which contributes to the activation gate at the S6 bundle crossing. IS6 also has L^1i22^, which contributes to the PyR2 site [12].

### 3.6. Allosteric Effects on Pyrethroid Action of kdr Mutations beyond the PyR1 and PyR2 Sites

Besides the kdr mutations within the PyR1 and PyR2 sites, multiple kdr mutations are located beyond the PyR1 and PyR2 sites and thus likely allosterically affect the action of pyrethroids (Table 3). The AF2-based models of AaNav1-1 allowed us to explore the contacts of the majority of respective WT residues and suggest mechanisms by which kdr mutations of respective residues allosterically affect the action of pyrethroids.

#### 3.6.1. M^827^I in the VSM-II Linker IIS1-S2

**Figure 5:**
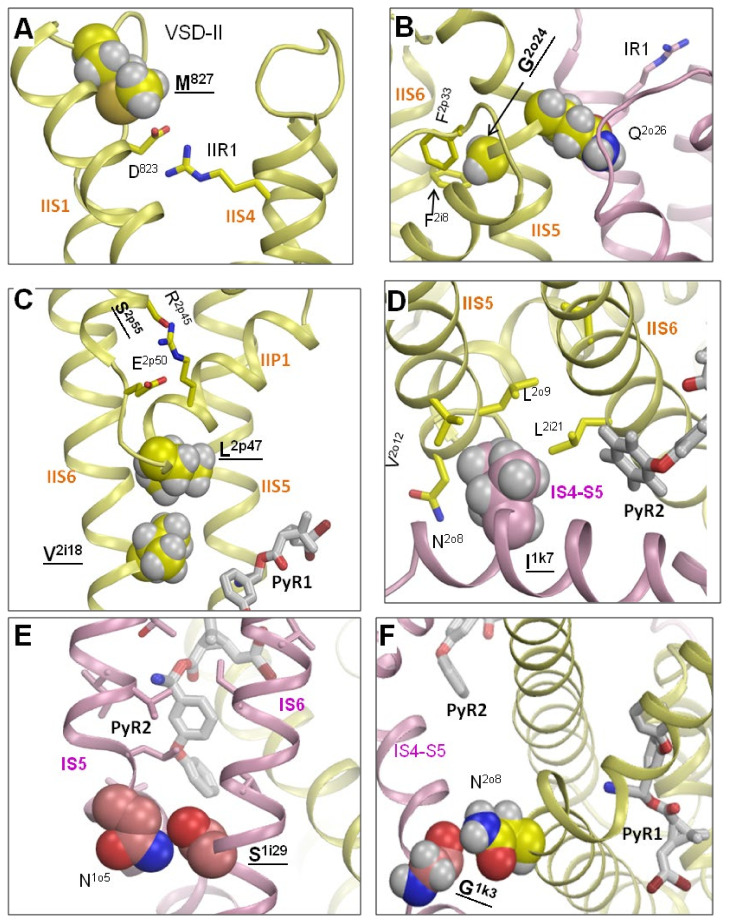
Residues beyond the PyR1 and PyR2 sites form intersegment contacts whose destruction would allosterically affect the action of pyrethroids. Labels of residues that are subject to kdr mutations are bold-underlined (see Section 3.6 for more detail). Panels (**A**–**F**) show, respectively, contacts of residues M^827^, G^2o24^, L^2p47/^S^2.p55^, I^1k7^, S^1i29^ and G^1k3^.

D^802^ in the BgNa_v_1-1a cockroach sodium channel is critical for the channel gating and action of pyrethroid insecticides [82]. In our recent study, D^802^ is proposed to form a salt bridge with the voltage-sensing arginine IIR1, the outermost arginine in IIS4 (Figure 5a), thus stabilizing the activated conformation of IIS4 [22]. Interestingly, as shown in Figure 5a, M^827^ approaches D^823^, which corresponds to D^802^ in BgNa_v_1-1a. The hydrophobic methyl group of M^827^ would strengthen the salt bridge D^823^:IIR1, while much shorter isoleucine M^827^I would not stabilize the salt bridge. As a result, the “up” conformation of IIS4, which is necessary for favorable interactions of M^2k11^ with DMT in the PyR1 site (Figure 4a), would be destabilized.

#### 3.6.2. G^943^A and Q^945^R in IIS6

Glycine G^943/2o24^ is involved in the knob-into-hole contact with F^2p33^ and F^2i8^ (Figure 5b), thus stabilizing the mutual disposition of helices IIS5 and IIS6. Mutation G^2o24^A would push away the extracellular ends of helices IIS5 and IIS6, which contribute, respectively, to the PyR1 and PyR2 sites.

Glutamine Q^945/2o26^ interacts with several residues in the extracellular loops of VSM-I (Figure 5b). Arginine Q^2o26^R would repel the voltage-sensing arginine IR1 and thus destabilize the “up” conformation of IS4 and linker-helix IS4-S5. The interaction of pyrethroids with the pyrethroid-sensing V^1k11^ [12,16] in the linker-helix IS4-S5 of the PyR2 site (Figure 4b) could be indirectly affected.

#### 3.6.3. S^989/2p55^P and D^1763^Y in P-Loops

Mutation S^989/2p55^P at the C-end of helix IIP2 is found only in combination with V^1016/2i18^G [83]. In the AF2 model, V^1016/2i18^ is involved in inter-segment contacts with L^2o9^, L^2o13^, and L^2p47^. These contacts stabilize the mutual disposition of helices IIS5 and IIS6, which contribute to PyR1, as well as to the geometry of loop IIP1-P2, with the selectivity-filter glutamate E^2p50^ (Figure 5c). Mutation V^1016/2i18^G eliminates these contacts. The destabilization of loop IIP1-P2 would affect the ion selectivity of the sodium channel [84]. Mutation S^989/2p55^P eliminates H-bond S^989/2p55^---R^2p45^, whereas released R^2p45^ would find another H-bonding partner. This may compensate for the impact of mutation V^1016/2i18^G on the ion selectivity (Figure 5c).

Mutation D^1763^Y in the extracellular loop IVS5-P1 is also found in combination with V^1016^^/2i18^G [61]. In the AF2 model, D^1763^ is salt-bridged with K^1774^ at the C-end of loop IVS5-P1. Elimination of the salt bridge in mutant D^1763^Y would affect the stability of the IVP1-P2 region, which contributes to the selectivity filter. The fact that two mutations, S^989^P and D^1763^Y, which are far beyond PyR1 or PyR2, are found in combination with V^1016/2i18^G, suggests that the double mutations may compensate for possible alterations in channel functional properties associated with individual mutations, which could cause some fitness issues in mosquitoes carrying these individual mutations.

#### 3.6.4. F^1538/3i17^L, D^1549/3i28^V, and E^1553/3i32^G in IIIS6

**Figure 6:**
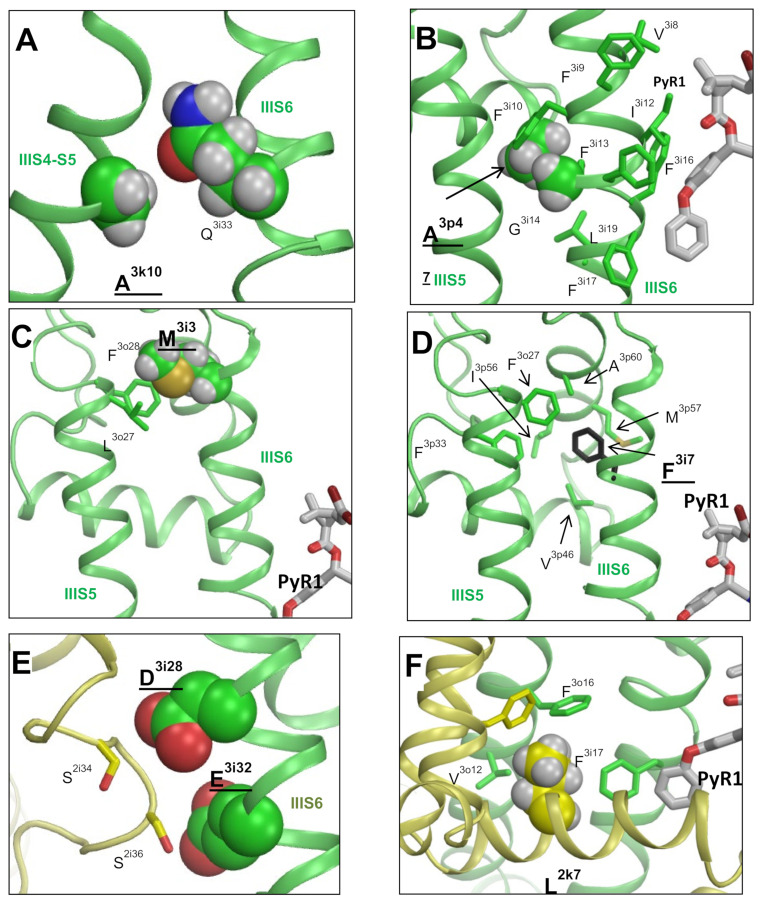
Residues beyond the PyR1 and PyR2 sites form intersegment contacts whose destruction would allosterically affect the action of pyrethroids. Labels of residues that are subject to kdr mutations are bold-underlined (see Section 3.6 for more detail). Labels of residues, which are not subject of kdr mutations, but to their engineered substitutions, are explored in functional studies and are bold-typed. (see Section 3.8 for more detail). Panels (**A**–**F**) show, respectively, contacts of residues A^3k10^, A^3p4^, M^3i3^, F^3i7^, D^3i28^/E^3i32^ and L^2k7^.

Phenylalanine F^3i7^ is involved in multiple hydrophobic contacts with P-loop residues in helices IIIP1 and IIIP2, thus stabilizing the P-loop domain (Figure 6d). Double mutation F^1538/3i7^L + M^4i3^I would destabilize this domain and affect the position of helix IIIS6, which contributes to the PyR1 site.

Glutamate E^1553/3i32^ forms an H-bond with S^2i36^ (Figure 6e). One helical turn above, D^1549/3i28^ is close to S^2i34^. Although an H-bond D^1549/3i28^---S^2i34^ is not seen in the ^i^AaNav1-1 model, its formation is possible upon rotation of the serine side chain, which lacks any H-bonding partners. The double mutation D^1549/3i28^V + E^1553/3i32^G, which has been detected in pyrethroid-resistant natural populations of *Helicoverpa armigera,* would destroy the inter-domain contact at the cytoplasmic end of PM, increasing the flexibility of helices IIS6 and IIIS6, which contribute, respectively, to the PyR2 and PyR1 sites.

Thus, the AF2 model suggests mechanisms by which most of the kdr mutations listed in Table 3 affect the geometry of the PyR1 or/and PyR2 sites. However, for several kdr mutations located in the cytoplasmic segments, which are unresolved in the AF2 model, the allosteric mechanisms of action on the PyR1 or PyR2 sites remain unclear.

### 3.7. Engineered Substitutions Not Reported as kdr Mutations

The effects on the channel gating and sensitivity to pyrethroids of many residue substitutions from the systematic mutational analysis of insect sodium channels have been explored in functional studies (Table 2 and Table 4). While these mutations have not been observed as kdr mutations, functional studies have shed additional light on the mechanisms of the channel gating and sensitivity to pyrethroids. The AF2-based models also allowed us to further rationalize observed effects of some engineered mutations within the PyR1 and PyR2 sites (Table 2), as well as beyond the two sites (Table 4).

The Kv1.2-based model of the open AaNav1-1 channel suggested that I^1k7^ contributes to the PyR2 site; mutation I^1k7^A facilitated activation and inactivation of the AaNav1-1 channel and strongly decreased its sensitivity to DMT and PMT [11]. In the AF2-based model ^o^AaNav1-1, I^1k7^makes tight hydrophobic inter-domain contacts with four residues (Figure 5d). DMT in the PyR2 site approaches I^1k7^, but it falls short of making direct contact with it. Mutation I^1k7^A would affect mutual disposition of helices IS4-S5, IIS5, and IIS6, which contribute to PyR1 and PyR2. Mutation I^1k7^A would also release contacts of the linker helix IS4-S5 with IIS5 and affect the channel gating. Facilitated inactivation would decrease the channel sensitivity to pyrethroids, as observed in previous experiments [11].

In the rotationally symmetric position of the open channel model, L^2k7^ is in tight hydrophobic contact with V^3o12^ and F^3o16^ in IIIS5 and F^3i17^ in IIIS6 (Figure 6f). Mutations L^2k7^F/I strongly facilitate activation and inactivation of the sodium channel from *D. melanogaster* [68]. These mutations would affect mutual disposition of helices IIS4-S5, IIS5, and IIIS6, which contribute to PyR1. This may explain why these mutations decrease the channel sensitivity to DMT [68].

Serine S^1i29^ is rather far from the PyR1-bound DMT, but mutation S^1i29^A strongly affects the channel sensitivity to PMT and DMT [12]. In model ^o^AaNav1-1, S^1i29^ forms an intersegment H-bond with N^1o5^ (Figure 5e). Mutation S^1i29^A eliminates this H-bond and would affect mutual disposition of helices IS6 and IS5, both of which contribute to the PyR1 site.

Substitutions D^823^G/A/K, but not D^823^E, would eliminate the salt bridge of D^823^ with R1 in VSM-II, which stabilizes the “up” state of the voltage-sensing helix IIS4 [22]. This would destabilize the “up” conformation of IIS4 and the IIS4-S5 linker in which M^2k11^ interacts with PMT (Figure 1c) and DMT (Figure 2a,c). Destabilization of the IIS4-activated conformation would separate M^2k11^ from the pyrethroids (Figure 4a) and thus decrease their action, as demonstrated experimentally [82].

An H-bond between the side chain of N^2o8^ and the backbone carbonyl of G^1k3^ (Figure 5f) stabilizes the mutual disposition of helices IIS4-S5 and IIS5, which contribute to the PyR1 site. This may explain why engineered mutation N^2o8^I decreased the action of DMT. It should be noted that N^2o8^I, as well as L^k7^F/I, which do not form direct contacts with pyrethroids, strongly decreased the action of DMT, but not PMT [68]. In view of our models, DMT is more sensitive than PMT to the mutations-associated deformations in the PyR1 site because the nitrile group of DMT makes an additional contact with T^2o10^ in the PyR1 site (Figure 2a,b). The fact that C^2i14^ makes specific contacts with DMT in both of its binding orientations (Figure 2a,c), but not with PMT (Figure 1c), explains why mutation C^2i14^A decreases the channel sensitivity to DMT, but not to PMT [68].

### 3.8. Cryo-EM Structure of NavPaS vs. AF2 Model of AaNav1-1

Earlier, the cryo-EM structure [28] was used to visualize the location of some pyrethroid-sensing residues in the PyR1 site of the mosquito sodium channels [87]. The overall folding of the NavPaS channel and the AF2 model ^i^AaNav1-1 is similar, but not identical (Figure S2). While the NavPaS structure greatly advanced our understanding of the sodium channel structure, the AF2-based models of insect sodium channels appear more appropriate for analyzing pyrethroid action because (i) the NavPaS is a non-functional channel due to unknown cause(s), (ii) The AF2 models predicted many specific inter-residue contacts beyond the PyR1 and PyR2 sites (Figure 5 and Figure 6), which explain the allosteric effects of respective mutations, and (iii) the AF2 models show structures of some segments that are unresolved in the NavPaS structure. While these segments do not contribute to the PyR1 or PyR2 sites, they seem important for predicting the channel states with the open PM and deactivated VSMs. The in silico transformations of the AF2 model in this study allowed us to understand the structural causes of the state-dependent action of pyrethroids.

### 3.9. Limitations of Our Modeling Approach

Molecular dynamics simulations and molecular modeling with Monte Carlo energy minimizations, which employ high-resolution crystal or cryo-EM structures of the sodium channels, are two major approaches in theoretical studies aimed to understand atomic details of interactions of these large transmembrane proteins with drugs and toxins, e.g., the work described in [88,89,90,91]. AF2-based models and in silico transformed models are not precise enough to unambiguously predict the lowest-energy ligand-binding poses. Furthermore, our computations ignore entropy, do not use explicit lipid and water molecules, and employ a rather simple treatment of electrostatic interactions. Among multiple low-energy binding poses found in the PyR1 and PyR2 sites, we presented those where pyrethroids form the maximal number of contacts with experimentally known pyrethroid-sensing residues. Energy characteristics of ligand–channel complexes are just approximate estimates of the enthalpy contribution to the free energy of ligand binding in a specific pose. Nevertheless, our results, which are obtained without any biasing of the ligand contacts with specific residues, are consistent with multiple sets of experimental data. The AF2-based models advance our understanding of how the binding of pyrethroids within and beyond the PyR1 and PyR2 sites stabilizes the activated channel conformation.

## 4. Conclusions

Our computations, which employed the AF2 neural network and in silico transformation of the channel structures, demonstrate that the majority of kdr and engineered mutations are located within the PyR1 and PyR2 sites. The models also suggest mechanisms by which mutations beyond the PyR1 and PyR2 sites affect the action of pyrethroids. Our study resolved the long-standing question regarding why pyrethroids preferably bind to activated channels and stabilize the channels in the activated state. Our models show that pyrethroids lose or weaken contacts with some pyrethroid-sensing residues in PyR1 and PyR2 upon the pore domain transition from the open to inactivated state and upon the transition of voltage-sensing helices in VSM-I and VSM-II from the activated (up) to deactivated (down) positions. The bound pyrethroids resist these transitions, thus stabilizing the channel in the activated state. We believe that the proposed models will be useful for the structure-based design of novel pyrethroids.

## Figures and Tables

**Table 1:** Kdr mutations in the PyR1 and PyR2 sites of insect sodium channels.

Mutation ^1^	Location	Species	Reference	Figure
V^253/1k11^F	PyR2	*Aedes aegypti*	[16]	2b,d
T^267/1o13^A	PyR2	*Plant hopper*	[17]	2b,d
V^410/1i19^M	PyR2	*Helicoverpa zea*	[32]	2b,d
V^410/1i19^L	PyR2	*Cimex luctularis*	[33]	2b,d
V^410/1i19^G/A	PyR2	*Helicoverpa zea*	[32]	2b,d
V^1010/2i12^L + L^1014/2i16^S	PyR2 + PyR2	*Anopheles culicifacies*	[34]	2b,d
I^1011/2i13^M	PyR2	*Aedes aegypti*	[35]	2b,d
I^1011/2i13^V	PyR2	*Aedes aegypti*	[36]	2b,d
N^1013/2i15^S	PyR2	*Anopheles sinensis*	[37]	2b,d
L^1014/2i16^F	PyR2	*Anopheles gambiae*	[38]	2b,d
L^1014/2i16^S	PyR2	*Anopheles arabiensis*	[39]	2b,d
L^1014/2i16^H	PyR2	*Helicoverpa zea*	[32]	2b,d
L^1014/2i16^C	PyR2	*Anopheles sinensis*	[40]	2b,d
L^1014/2i16^W	PyR2	*Anopheles sinensis*	[37]	2b,d
L^1014/2i16^F + F^979^S	PyR2 + IIP1	*Myzus persicae*	[41]	2b,d
L^1014/2i16^F + N^1575^Y	PyR2 + III-IV	*Anopheles gambiae*	[42]	2b,d
L^1014/2i16^F+E435K+C785R	PyR2	*Blattella germanica*	[43]	2b,d
M^918/2k11^T + L^1014/2i16^F	PyR1 + PyR2	*Haematobia i. irritans*	[44]	1c, 4a,b,d
M^918/2k11^L + V^1010/2i12^A	PyR1 + PyR2	*Thrips tabaci*	[45]	1c, 2a
M^918/2k11^I + L^1014/2i16^F	PyR1 + PyR2	*Plutella xylostella*	[46]	1c, 2a,b,d
T^929/2o10^I + L^1014/2i16^F	PyR1 + PyR2	*Frankliniella occidentalis*	[47]	1c, 2a,b,d
T^929/2o10^I + L^1014/2i16^F	PyR1 + PyR2	*Plutella xylostella*	[48]	1c, 2a,b,d
T^929/2o10^C + L^1014/2i16^F	PyR1 + PyR2	*Frankliniella occidentalis*	[47]	1c, 2a
T^929/2o10^V + L^1014/2i16^F	PyR1 + PyR2	*Ctenocephalides felis*	[49]	1c, 2a
T^929/2o10^N + L^1014/2i16^F	PyR1 + PyR2	*L. decemlineata*	[50]	1c, 2a,b,d
F^979/2p44^S + L^1014/2i16^F	PyR1 + PyR2	*Myzus persicae*	[41]	1c, 2a,b,d
M^918/2k11^T	PyR1	*Aphis gossypii*	[51]	1c, 2a
M^918/2k11^L	PyR1	*Aphis gossypii*	[52]	1c, 2a
M^918/2k11^L + L^925/2o6^I	PyR1 + PyR1	*Trialeurodesvaporariorum*	[53]	1c, 2a
M^918/2k11^V	PyR1	*Bemisia tabaci*	[54]	1c, 2a
L^925/2o6^I	PyR1	*Bemisia tabaci*	[54]	1c, 2a
L^925/2o6^V	PyR1	*Varroa destructor*	[55]	1c, 2a
T^929/2o10^I	PyR1	*Thrips tabaci*	[56]	1c, 2a
T^929/2o10^I + L^932/2o13^F	PyR1 + PyR1	*P. humanus capitis*	[57]	1c, 2a
T^929/2o10^I + M^827^I	PyR1 + VSM2	*P. humanus capitis*	[58]	1c, 2a
T^929/2o10^C	PyR1	*Frankliniella occidentalis*	[47]	1c, 2a
T^929/2o10^V	PyR1	*Bemisia tabaci*	[59]	1c, 2a
L^932/2o13^F + M^827^I	PyR1 + VSM2	*P. humanus capitis*	[58]	1c, 2a
I^936/2o17^V	PyR1	*Helicoverpa zea*	[32]	1c, 2a
L^982/2p47^W	PyR1	*Aedes aegypti*	[35]	1c
V^1016/2i18^G	PyR1	*Aedes aegypti*	[35]	2a,c
V^1016/2i18^G + S^989/2p55^P	PyR1	*Aedes aegypti*	[60]	2a,c
V^1016/2i18^G + D^1763^Y	PyR1	*Aedes aegypti*	[61]	2a,c
V^1016/2i18^I	PyR1	*Aedes aegypti*	[36]	2a,c
F^1020/2i22^S	PyR1	*Blattella germanica*	[62]	2a
L^1024/2i26^V	PyR1	*Tetranychus urticae*	[63]	1c, S5b
F^1534/3i13^C	PyR1	*Aedes aegypti*	[64]	1c, 2a
F^1534/3i13^L	PyR1	*Aedes aegypti*	[65]	1c, 2a
F^1537/3i16^L	PyR1	*Dermanyssus gallinae*	[66]	1c
F^1538/3i17^I	PyR1	*Rhipicephalus microplus*	[67]	1c, 2a

^1^ Each mutation is labeled with the position of the residue in the house fly sodium channel (GenBank accession number: X96668), followed by another label using the universal labeling system for P-loop channels, as explained in the Figure 1 legend.

**Table 2:** Engineered mutations within PyR2 and PyR1 that affect the action of pyrethroids.

Mutation ^1^	Location	Channel	Reference	Figure
L^260/1o6^A	PyR2	AaNa_v_1-1	[12]	2b,d
I^264/1o10^C	PyR2	AaNa_v_1-1	[11]	2b,d
I^413/1i22^A	PyR2	AaNa_v_1-1	[12]	2b,d
M^918/2k11^T	PyR1	DmNa_v_	[68]	4c,2a,c
V^922/2o3^I ^a^	PyR1	BiNa_v_1-1	[15]	6c
L^925/2o6^I	PyR1	DmNa_v_	[68]	1c
T^929/2o10^ I	PyR1	DmNa_v_	[68]	1c,2a
L^932/2o13^F	PyR1	DmNa_v_	[68]	1c,2a
C^933/2o14^A	PyR1	DmNa_v_	[68]	1c,2a
I^936/2o17^V	PyR1	DmNa_v_	[68]	1c,2a
N^1013/2i15^S	PyR2	AaNa_v_1-1	[12]	2b,d
F^1020/2i22^S	PyR1	AaNa_v_1-1	[12]	1c,2a
L^1023/2i25^A	PyR1	AaNa_v_1-1	[12]	2a
L^1024/2i26^A	PyR1	AaNa_v_1-1	[12]	1c
F^1526/3i5^L	PyR1 (*τFVL*)	BiNa_v_1-1	[15]	6c
V^1529/3i8^A	PyR1 (*τFVL*)	BiNa_v_1-1	[15]	1c,2a
I^1533/3i12^A	PyR1	BgNa_v_1-1a	[69]	1c,2a
F^1537/3i16^A	PyR1	BgNa_v_1-1a	[69]	2a

^a^ M^2o3^ in AaNav1-1a. ^1^ Each mutation is labeled with the position of the residue in the house fly sodium channel (GenBank accession number: X96668), followed by another label using the universal labeling system for P-loop channels, as explained in the Figure 1 legend.

**Table 3:** Kdr Mutations beyond the PyR1 and PyR2 sites.

Mutation ^1^	Species	Reference	Impact	Figure
D^59^G	*Blattella germanica*	[43]		
A^99^S	*Culex quinquefasciatus*	[75]		
I^254/1k12^N	*Drosophila melanogaster*	[76]		
E^435/1i45^K	*Blattella germanica*	[43]		
C^785^R	*Blattella germanica*	[43]		
M^827/IIS1-S2^I	*P. humanus capitis*	[58]	PyR1	5a
G^943/2o24^A	*P. humanus capitis*	[77]	PyR1, PyR2	5b
Q^945/2o26^R	*L. salmonis*	[78]	PyR2	5b
S^989/2p55^P + V^1016/2i18^G	*Aedes aegypti*	[60]	IIP1-P2 + PyR1	5c, 2a,c
A^1101^T + P^1879/CTD^S	*Plutella xylostella*	[46]	II/III + III/IV	
A^1410/3k10^V	*Drosophila melanogaster*	[76]	PyR1	6a
A^1494/3p47^V	*Drosophila melanogaster*	[76]	PyR1	6b
M^1524/3i3^I	*Drosophila melanogaster*	[76]	PyR1	6c
F^1528/3i7^L + M^1823/4i3^I	*Varroa destructor*	[79]	PyR1 + IVS6	6d
D^1549/3i28^V + E^1553/3i32^G	*Helicoverpa armigera*	[80]	PyR1, PyR2	6e
A^1215^D + F^1538/3i17^I	*Tetranychus urticae*	[81]	II/III + PyR1	
W^1594^R	*Culex quinquefasciatus*	[75]	N-end of IVS0	

^1^ Each mutation is labeled with the position of the residue in the house fly sodium channel (GenBank accession number: X96668), followed by another label using the universal labeling system for P-loop channels, as explained in the Figure 1 legend.

**Table 4:** Engineered mutations beyond PyR1 and PyR2 that affect the action of pyrethroids.

*Mutation ^a^*	Species	Reference	Impact	Figure
I^249/1k7^A	*Aedes aegypti*	[11]	PyR2, PyR1	5d
S^420/1i29^A	*Aedes aegypti*	[12]	PyR1	5e
D^823^G/A/K	*Blattella germanica*	[82]	PyR1	5a
L^914/2k7^F/I	*Drosophila melanogaster*	[68]	PyR1	6f
N^927/2o8^I	*Drosophila melanogaster*	[68]	PyR1, PyR2	5f
A^1410/3k10^ V	*Blattella germanica*	[85]	PyR1	6a
A^1494/3p47^V	*Blattella germanica*	[85]	PyR1	6b
G^1535/3i14^A ^b^	*Blattella germanica*	[69]	PyR1	
N^1541/3i20^A	*Blattella germanica*	[69]	PyR1	
D^1549/3i28^V	*Blattella germanica*	[86]		6e

^a^ Each mutation is labeled with the position of the residue in the house fly sodium channel (GenBank accession number: X96668), followed by another label using the universal labelling system for P-loop channels, as explained in the Figure 1 legend. ^b^ Gating hinge in IIIS6.

## Data Availability

No experimental data were generated in this study.

## Supplementary Materials

The following are available online at https://www.mdpi.com/article/10.3390/insects13080745/s1. Figure S1: Sequence alignment of domains I-IV of the AaNav1-1 channel and list of models used in this work. Figure S2: Intra-membrane and extracellular views at the cryo-EM structure of cockroach non-functional sodium channel NavPaS and the AF2 model of the mosquito sodium channel AaNav1-1. Figure S3: AF2 models of the AaNav1-1 and BgNav1-1a channels. Figure S4: The AF2 model of the channel with inactivated PM overlaid over the channel model with in silico opened pore domain. Figure S5: Intersegment contacts of residues in the PyR1 site whose kdr mutations may exert dual impact on the action of pyrethroids.

## Abbreviations

AF2: AphaFold2; DMT, deltamethrin; kdr, knockdown resistance; MCM, Monte Carlo minimization; PM, pore module; PMT, permethrin; PyR1, pyrethroid receptor site 1; PyR2, pyrethroid receptor site 2; VSM, voltage-sensing module; WHO, World Health Organization.
