# Supplementary material for: Pyrethroids in an AlphaFold2 Model of the Insect Sodium Channel
Source: Insects. 2022 Aug 18;13(8):745. doi: 10.3390/insects13080745 (PMC9409284; doi:10.3390/insects13080745)
Supplement: Supplementary file 1 [file insects-13-00745-s001.zip › insects-1844594-supplementary.pdf]

Supplemental Figures

Pyrethroids in the AlphaFold2 Model of Insect Sodium Channel

Boris S. Zhorov[1,2] and Ke Dong[3]

[1]Department of Biochemistry and Biomedical Sciences, McMaster University, Hamilton L8S 4K1 Canada;
[2] Sechenov Institute of Evolutionary Physiology & Biochemistry, Russian Academy of Sciences, St. Petersburg, 194223
[3] Department of Biology, Duke University, Durham, NC 27708, U.S.A.

Segment		#	k1	k11	o1	o11	o21
I	L45-S5	243	VPGLKTIVGA	VI	ESVKNLRDVI	ILTMFSLSVF	ALMGLQIY
II	L45-S5	908	WPTLNLLISI	MG	RTMGALGNLT	FVLCIIIFIF	AVMGMQLF
III	L45-S5	1401	MQGMRVVVNA	LV	QAIPSIFNVL	LVCLIFWLIF	AIMGVQLF
IV	L45-S5	1719	AKGIRTLLFA	LA	MSLPALFNIC	LLLFLVMFIF	AIFGMSFF
			p37	p41	p51	p61	
I	P	363	GWAF	LSAFRLMTQD	YW_ENLYQLV	LRS	
II	P	972	MHSF	MIVFRVLCGE	_WIESMWDCM	LVG	
III	P	1484	GKAY	LCLFQVATFK	GW_IQIMNDA	IDS	
IV	P	1777	GQSM	ILLFQMSTSA	GW_DGVLDGI	INE	
			i1	i11	i21		
I	S6	392	PWHMLFFIVI	IFLGSFYLVN	LILAIVAMSY		
II	S6	999	VSCIPFFLAT	VVIGNLVVLN	LFLALLLSNF		
III	S6	1522	IYMYLYFVFF	IIFGSFFTLN	LFIGVIIDNF		
IV	S6	1822	TIGITYLLAY	LVISFLIVIN	MYIAVILENY		

Figure S1. Sequence alignment of linker-helices L45 (S4-S5), outer helices S5, P-loops and inner helices S6 in repeats I-IV of the AaNav1-1 channel. Top rows above the sequences show residue labels, which are universal for P-loop channels. Column "#" shows the housefly number for the leftmost residue in the row.

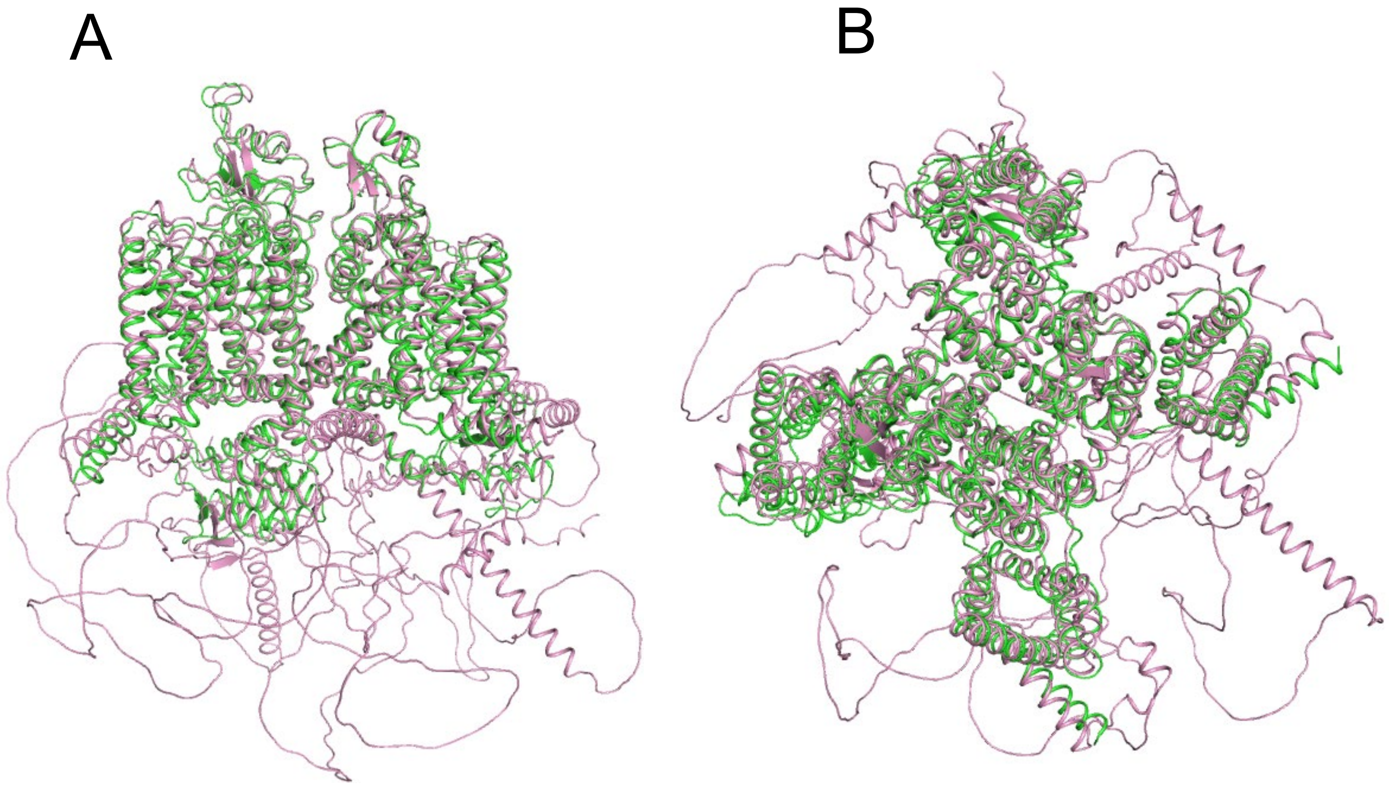

Figure S2. Intra-membrane (**A**) and extracellular (**B**) views at the cryo-EM structure of cockroach non-functional sodium channel NavPaS (green, PDB ID: 5X0M) and the AlphaFold2 model of the mosquito sodium channel AaNav1-1 (magenta). The structures were superimposed by minimizing RMS deviations of the four P1 helices, which are the most structurally conserved segments of P-loop channels against the crystal structure of Kv1.2/Kv2.1 channel (PDB ID: 2R9R). Folding of extracellular and transmembrane segments is very similar. The AF2 model has several structured segments that are not resolved in the cryo-EM structure of NavPaS or other eukaryotic sodium channels.

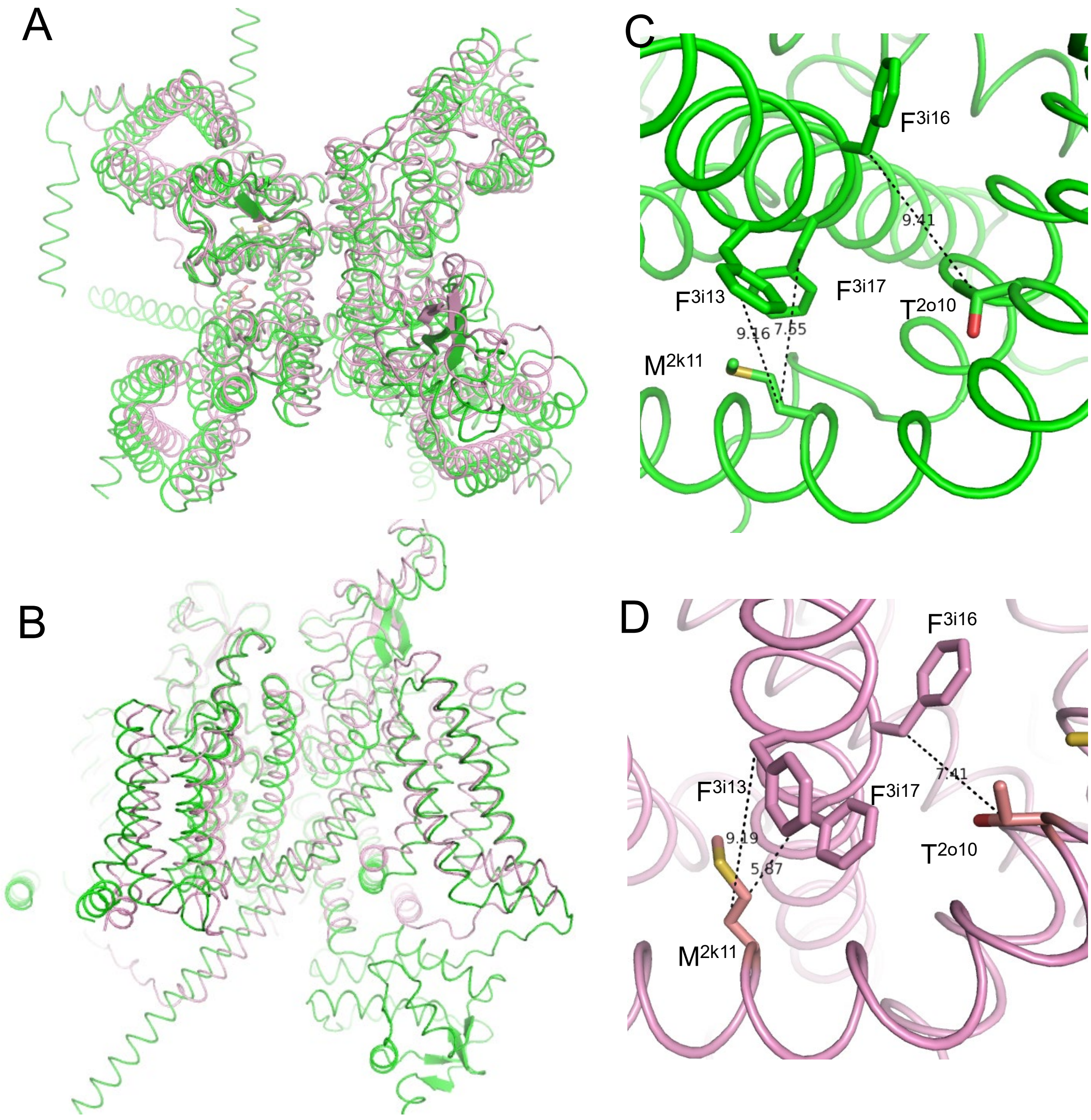

Figure S3. AlphaFold2 (green) and RoseTTAFold (pink) models of the AaNav1-1 channel 3D-aligned with Chimera-X (Pettersen et al., 2021). In the AF2 model unstructured cytoplasmic loops are removed. In the RoseTTAFold model (Baek et al., 2021), the N-terminal part and C-terminal domain are removed and large cytoplasmic linkers are replaced with 20-residue glycine linkers to satisfy the size limitation of the Robetta Server (http://robetta.bakerlab.org) and preserve a single-subunit sequence of the model. Despite the different size of the models, the overall folding of the transmembrane and extracellular parts is rather similar. **A, B.** Extracellular and intermembrane views. **C, D.** Enlarged views of the PyR1 site in the models. Distances between CB atoms of pyrethroid-sensing residues in the AF2 model is larger than in the RoseTTAFold model.

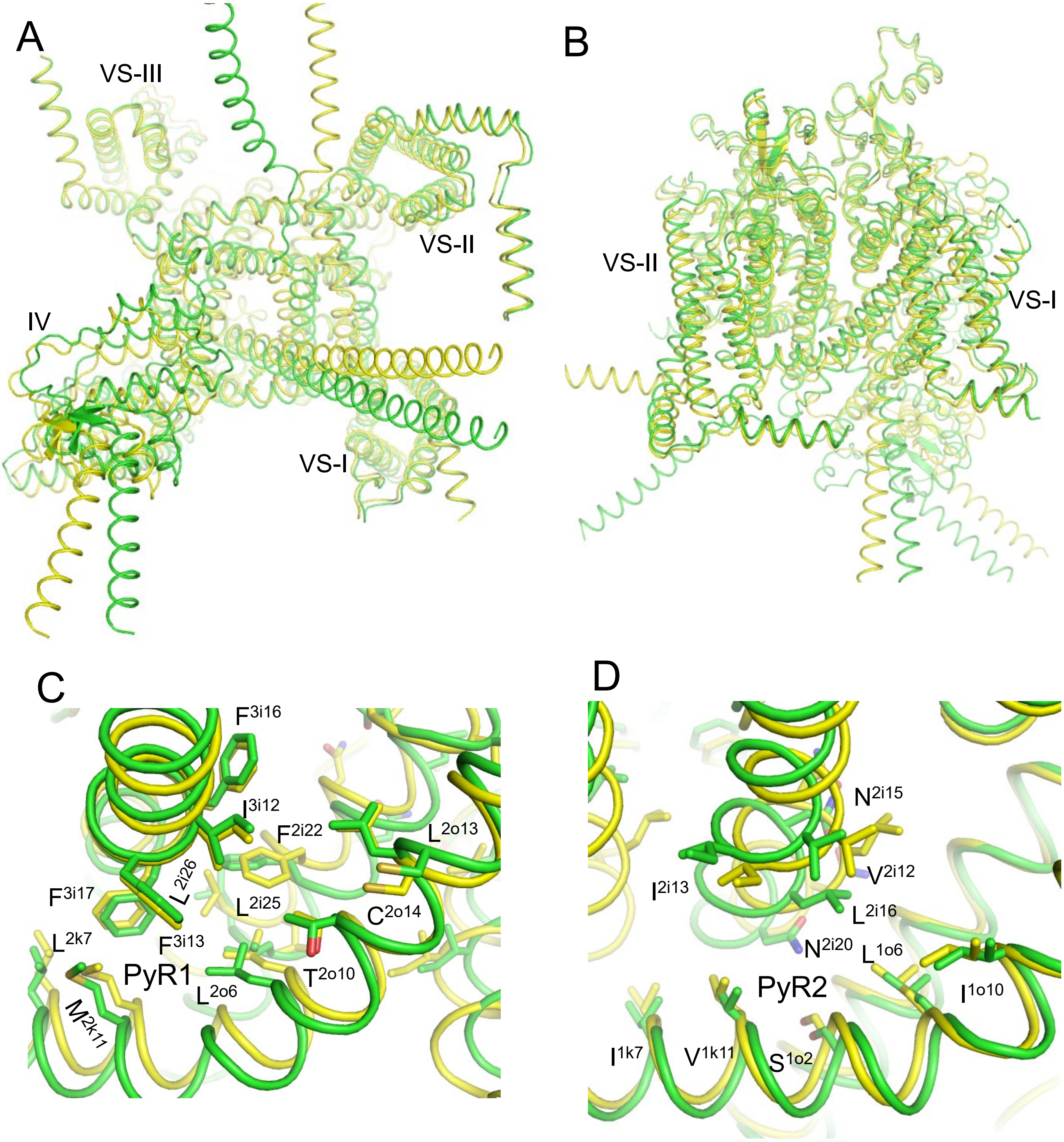

Figure S4 The AF2 model of the channel with inactivated PD (^{i}AaNav1-1, yellow) overlaid over the channel model with *in-silico* opened PD (^{i}AaNav1-1, green). **A, B.** Extracellular and intermembrane views. **C, D.** Enlarged views of the PyR1 and PyR2 sites in the models. Note a large shift of helix IIS6 in which several residues contribute to the PyR2 site.

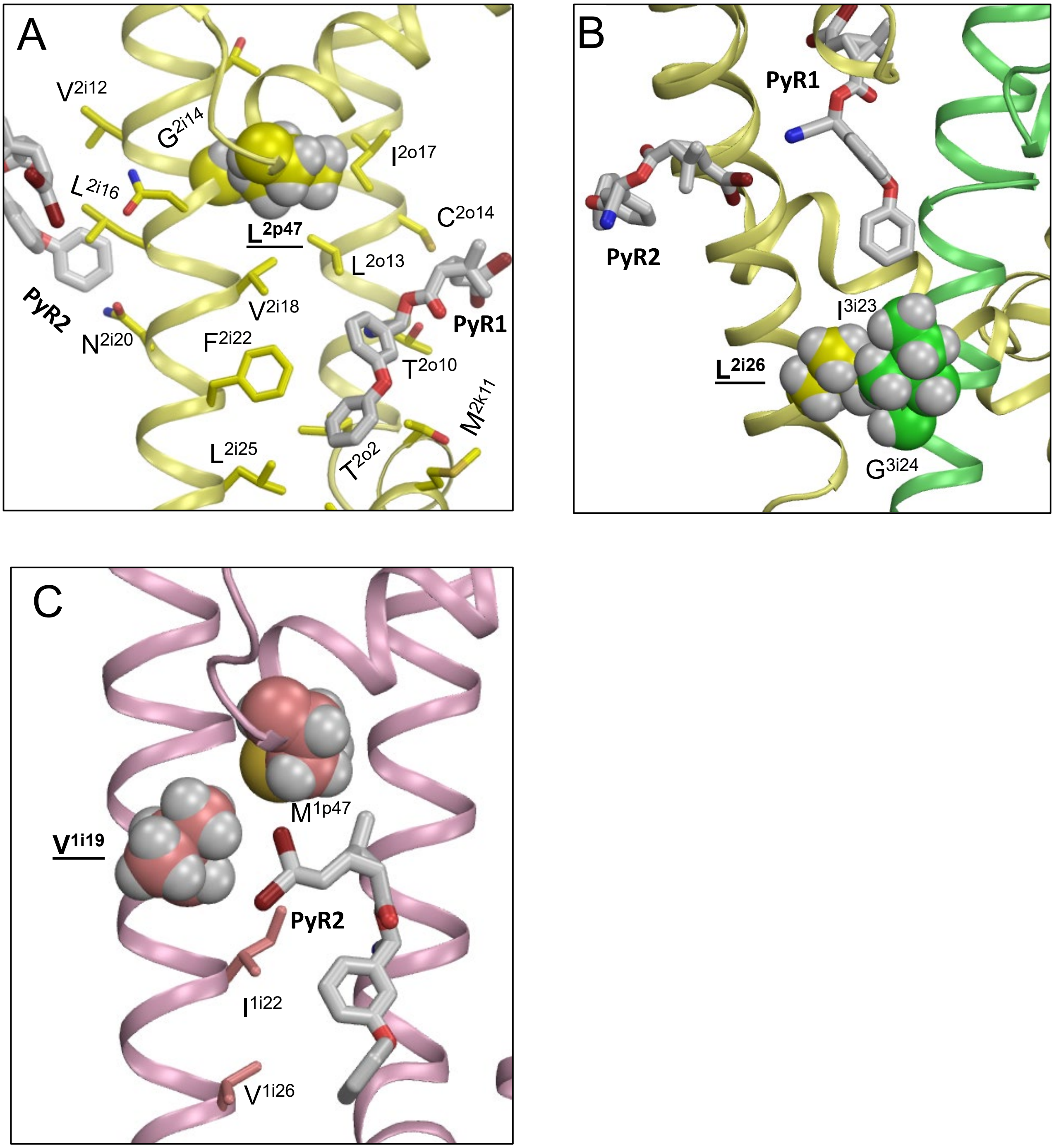

Figure S5. Intersegment contacts of residues in the PyR1 site whose kdr mutations may exert dual impact on the action of pyrethroids (see Section 3.11 for mode detail).
